# Genome-wide analysis of LysM gene family members and their expression in response to *Colletotrichum fructicola* infection in Octoploid strawberry(*Fragaria* × *ananassa*)

**DOI:** 10.3389/fpls.2022.1105591

**Published:** 2023-01-23

**Authors:** Liqing Zhang, Shuigen Li, Xianping Fang, Haishan An, Xueying Zhang

**Affiliations:** Shanghai Key Laboratory of Protected Horticultural Technology, Forestry and Fruit Tree Research Institute, Shanghai Academy of Agricultural Sciences, Shanghai, China

**Keywords:** *Fragaria* × *ananassa*, LysM protein, genome-wide analysis, defense response, chitin

## Abstract

The cultivated octoploid strawberry (*Fragaria* × *ananassa*) is an economically important fruit that is planted worldwide. The lysin motif (LysM) protein family is composed of the major class of plant pattern recognition receptors, which play important roles in sensing pathogen-associated molecular patterns (PAMPs), and subsequently triggers downstream plant immunity. In the present study, a comprehensive, genome-wide analysis of *F*. × *ananassa LysM* (*FaLysM*) genes was performed to investigate gene structures, phylogenic relationships, chromosome location, collinear relationships, transcription factor binding sites, and protein model analysis. We aimed to identify the *LysM* genes involved in the defense against plant pathogens. A total of 14 *FaLysM* genes were identified in the *F*. × *ananassa* genome and divided into 2 subgroups (LYP and LYK) on the basis of the phylogenetic analysis. The Ka/Ks ratio for the duplicated pair of most *FaLysM* genes was less than 1, which indicates that the selection pressure was mostly subject to the purifying selection during evolution. The protein model analysis revealed that FaLysM2-10 contain conserved mode of chitin binding, which suggest the potential role of FaLysM2-10 in pathogen perception and plant immunity. The RNA-Seq results showed the differential regulation of 14 *FaLysM* genes in response to *Colletotrichum fructicola* infection, implying the complex interaction between *C*. *fructicola* and strawberry. Knockout of candidate effector gene *CfLysM2*, which was previously proved to be highly expressed during *C*. *fructicola* infection, resulted in the up-regulation of six *FaLysM* genes (*FaLysM1*, *FaLysM2*, *FaLysM3*, *FaLysM7*, *FaLysM8*, and *FaLysM12*), indicating the competitive relations between *CfLysM2* and *FaLysM* genes. Overall, this study provides fundamental information on the roles of LysM proteins in octoploid strawberry and its interaction with *C*. *fructicola*, laying useful information for further investigation on the *C*. *fructicola*-strawberry interaction and strawberry resistance breeding.

## Introduction

Throughout their lifetimes, plants are constantly challenged by pathogens. During evolution, a complex and close relationship is developed between plants and pathogens, with infections and defenses being the most crucial relationship. Microbe- (MAMPs) or pathogen-associated molecular patterns (PAMPs) such as fungal chitin, bacterial lipopolysaccharide, flagellin, and peptidoglycan (PGN) are conserved microbe-specific molecules recognized by pattern recognition receptors (PRRs) in the plant innate immune system, known as PAMP-triggered immunity (PTI) (Schwessinger and Zipfel, 2008). Correspondingly, pathogens produce effectors that inhibit the PTI and therefore become more virulent, a state known as effector-triggered immunity (ETI) (Boller and He, 2009). Recent studies have shown that ETI and PTI have a symbiotic crosstalk, which plays a potential interconnected role to create a robust plant defense system (Yuan et al., 2021).

According to the characteristic motifs in the extracellular regions, representative MAMP receptors are classified into distinct subfamilies, such as the leucine-rich repeat (LRR) and lysin motif (LysM) domain–containing receptors (Boutrot and Zipfel, 2017; Saijo et al., 2018). LysM, which contains a highly conserved βααβ secondary structure responsible for the binding of chitin and peptidoglycans (Gust et al., 2012), was first found in *Bacillus phage* Φ29 lysozyme (Garvey et al., 1986), and then proved to be a ubiquitous protein motif in almost all organisms (Buist et al., 2008). The LysM protein family harbor a variable number of tandem LysM domains (1 to 3). For plant lineage, four types of LysM family proteins are involved: LysM-containing receptor-like kinase (LYK), LysM-containing receptor like protein (LYP), extracellular LysM proteins (LysMe) and nonseretory intracellular proteins (LysMn) (Zhang et al., 2007). LYP and LYK which exist widely in the plant kingdom are relatively well studied among them (Kaku et al., 2006).

CEBiP (chitin elicitor-binding protein) is the first and currently the most clarified LysM protein family. *Oryza sativa* CEBiP (OsCEBiP) was first identified binding to chitin and subsequently inducing downstream immune responses (Kaku et al., 2006). A similar function of homologous CEBiP was proved in other plants, such as *Triticum aestivum* CEBiP (TaCEBIP) and *Hordeum vulgare* CEBiP (HvCEBIP) (Tanaka et al., 2010; Lee et al., 2014; Jian and Liang, 2019). Later, CERK1 (chitin elicitor receptor kinase; also known as LysM-RLK1), the interaction partner of CEBiP, was identified. The knockout of *Arabidopsis CERK1* (*AtCERK1*) completely deprives responses to the chitin elicitor, such as mitogen-activated protein kinase MAPK activation and reactive oxygen species production (Miya et al., 2007, Petutschnig et al., 2010). *Oryza sativa* CERK1 (OsCERK1) directly interacts with OsCEBiP and forms a receptor complex to cooperatively regulate chitin elicitor signaling (Shimizu et al., 2010). Except for the perception of fungal chitin, the plant LysM protein family was also identified as a bacterial peptidoglycan (PGN) receptor. *Arabidopsis* LYM1 and LYM3 (also known as LYP1 and LYP3, respectively) are two plasma membrane proteins that directly interact with PGN and mediate the immunity to bacterial infections (Willmann et al., 2011). Some members of the LysM protein family play dual roles in both chitin and PGN perceptions. For example, two rice LysM proteins, OsLYP4 and OsLYP6, were identified as the receptors of both chitin and PGN as responsible for pathogen-induced defense (Liu et al., 2012a; Liu et al., 2013). Besides being the receptor mediating the immune response to fungal and bacterial infections, the plant LysM protein family also functions in plant-microorganism symbiotic interactions. For example, *Pisum sativum* LYK9 (PsLYK9) can sense the long- and short-chain oligosaccharides of fungi and initiate immune response. On the other hand, PsLYK9 also plays a role in symbiosis development with arbuscular mycorrhizal (AM) fungi (Leppyanen et al., 2021). Similarly, *Solanum lycopersicum* LYK10 (SlLYK10) and LYK12 (SlLYK12) were also found to be involved in the regulation of AM symbiosis (Buendia et al., 2016; Liao et al., 2018).

The LysM protein can act as a pattern recognition receptor on the cell membrane, transmitting signals to the plant cells and triggering PTI. Moreover, it can also act as an effector to help pathogens penetrate plants, inducing ETI. So far, the most clarified LysM-containing effector is Ecp6, which efficiently binds chitin. Ecp6 was proved to perturb chitin-induced immunity. The probable mechanism is that Ecp6 sequesters chitin fragments to evade the perception by plant immune system (De Jonge et al., 2010; Sánchez-Vallet et al., 2013). *Magnaporthe oryzae* Slp1 was demonstrated to play a similar role in the inhibition of chitin-triggered immunity (Mentlak et al., 2012). However, unlike *Cladosporium fulvum* Avr4 and *Mycosphaerella graminicola* Mg1LysM and Mg3LysM, which can protect fungal hyphae from the hydrolytic activity of chitinases, Ecp6 and Slp1 have no such capacity (De Jonge et al., 2010; Marshall et al., 2011; Mentlak et al., 2012). These studies collectively proved the important role of the deregulation of chitin-triggered immunity by LysM effectors, albeit with varied mechanisms. The suppression of chitin-triggered immunity by LysM effectors has been speculated to be dependent on its ultrahigh affinity for chitin binding, outcompeting the substrate binding by plant chitin receptors such as the LysM protein family (Sánchez-Vallet et al., 2013; Sanchez-Vallet et al., 2015). Fungal LysM effectors are multifunctional proteins that occur in fungal species with different lifestyles, although chitin binding is their most well-known function reported so far. For example, the addition of the purified LysM effector to *Trichoderma atroviride* inhibits spore germination (Seidl-Seiboth et al., 2013). Our previous study identified 52 candidate fungal effector of *Colletotrichum fructicola* during its infection on strawberry leaves. Among them, *CfLysM2*, a LysM effector gene which is homologous to *Slp1*, was significantly upregulated at the onset of infection. Importantly, the pathogenicity was significantly attenuated after deletion mutation of *CfLysM2* (Zhang et al., 2018; Yu et al., 2022).

Strawberry (*Fragaria* × *ananassa* Duch.) is one of the most economically important horticultural crops worldwide. However, strawberry plants are prone to many diseases (Hsu et al., 2022). Pathogen-derived injuries or loss due to fungi, bacteria, and so on is a challenge for strawberry producers (Whitaker et al., 2020). Among strawberry diseases, anthracnose disease caused by *Colletotrichum fructicola* can have devastating effects on strawberry plants (Zhang et al., 2020). The genome of the cultivated octoploid strawberry is one of the most complex plant genomes. It originates from four different diploid ancestors and is an allopolyploid (Edger et al., 2019). The recently published octoploid strawberry genome sequence and transcriptome-wide analysis lay a solid foundation for the identification and analysis of the *LysM* gene family. At present, 15 *FvLysM* genes have been identified in wild strawberry (*F*. *vesca*) (Liu et al., 2020). Compared with wild strawberry, octoploid strawberry is more susceptible to bacterial and fungal diseases (Martínez Zamora et al., 2008). The LysM proteins are important PRRs involved in plant innate immunity. However, little information is available about the octoploid strawberry. In this study, a genome-wide analysis of *LysM* genes in the octoploid strawberry (*FaLysM* genes) was conducted, including classification, physicochemical properties, gene structure, phylogenetic evolution, chromosome location, collinear relationships, transcription factor (TF)-binding sites, and protein model analysis. Nine FaLysM proteins possessed the highly conserved mode, including seven conserved residues (labeled R1–R7) homologous to the OsCEBiP protein in rice. Furthermore, the expression patterns of the *FaLysM* genes after *C*. *fructicola* wild strain (WT) and effector gene *CfLysM2* knockout mutant (Δ*CfLysM2*) infection were profiled. *CfLysM2* was previously proved to be highly expressed during infection and contributed to the virulence of *C*. *fructicola* (Yu et al., 2022). The distinctive expression profiles of *FaLysM* genes were observed in strawberry leaves infected with *C*. *fructicola* WT and Δ*CfLysM2*, indicating the complex interaction between *C*. *fructicola* and strawberry. These findings provide a basis for further functional characteristics of FaLysM proteins in the octoploid strawberry and insights into the strategies for disease resistance breeding of strawberry.

## Materials and methods

### Sequence identification of the *LysM* gene family members in octoploid strawberry

The genome information of octoploid strawberry (*F*. × *ananassa*) was downloaded from the GDR database (https://www.rosaceae.org/species/fragaria_x_ananassa/genome_v1.0.a1). The corresponding LysM protein sequences were downloaded from the *Arabidopsis* database (TAIR; http://www.Arabidopsis.org/). The candidate LysM members in the octoploid strawberry were identified through a local BLASTP search using the known LysM proteins of *Arabidopsis thaliana* as a query. All LysM-containing sequences of the octoploid strawberry were further investigated using the hidden Markov model of the LysM domain (Pfam ID: PF01476) to search against the octoploid strawberry genome for the identification of candidates with *E* values < 1e-20. Pfam A (v33.1) and pfamscan (v1.6) database were used for domain validation.

The molecular weight, length, and theoretical isoelectric points of the LysM proteins in the octoploid strawberry were determined using ProtParam (https://web.expasy.org/protparam/), a bioinformatic tool from the ExPASY resource portal. The subcellular localization predictions of the LysM members were made using the website ProtComp 9.0 (http://www.softberry.com/berry.phtml?topic=protcomppl&group=programs&subgroup=proloc). The SignalP 4.0 server (http://www.cbs.dtu.dk/services/SignalP-4.0/) was used to perform the prediction analysis of protein signal peptides. The transmembrane domain predictions were made using WoLF PSORT (https://www.genscript.com/wolf-psort.html).

### Protein model analysis of FaLysM proteins with Phyre^2^ server

The models of the FaLysM proteins were structured using the web tool Phyre^2^ (http://www.sbg.bio.ic.ac.uk/phyre2). The amino acids of the FaLysM proteins were uploaded and aligned on the website. For the structural models, the confidence level for quality control and threshold was 100%. The predicted 3D structures were delivered through emails and downloaded as PDB files, which were further visualized and modeled using the molecular modeling tool PyMOL (Version 1.7.4) (DeLano, 2002).

### Phylogenetic analysis of and classification of LysM proteins

The LysM protein sequences of Arabidopsis (*Arabidopsis thaliana*, At), wild strawberry (*F*. *vesca*, Fv), soybean (*Glycine max*, Gm), rice (*Oryza sativa*, Os), and apple (*Malus domestica*, Md) were used to generate phylogenetic trees *via* MAFFT v7.427 multiple sequence alignments with the default parameters, the accession number of indicated LysM protein sequences was listed in **Supplementary Table S1**. A neighbor-joining (NJ) phylogenetic tree was constructed using the MEGA-X software (Kumar et al., 2018). The p distance was used and the optional parameters for pairwise deletion were considered.

### Structure and conserved motif analysis of *FaLysM* genes

The online tool GSDS 2.0 (http://gsds.gao-lab.org/) was used to display *FaLysM* gene structures based on their genomic sequences and coding regions, including exon and intron numbers and lengths (Hu et al., 2015). A conserved motif analysis of the LysM proteins of octoploid strawberry was performed using online Multiple Expectation Maximization for Motif Elicitation (https://meme-suite.org/meme/tools/meme), as previously described (Bailey et al., 2015). The parameters were arbitrary repetitions, and the maximum motif numbers were 15.

### Physical localization, collinearity analysis, and Ka/Ks calculation of duplicated *FaLysM* genes

By using MapChart 2.3, the genome annotation of all *FaLysM* genes was mapped onto the chromosomes to identify their physical locations. The Multiple Collinearity Scan toolkit (MCScanX, gap_penalty: −1, *E* = 1e-10) was used to analyze the collinearity of intra- and inter-specific genes, and the results were visualized using a Circos multiple synteny plot, in which the interspecies were *F*. *vesca* and *Malus domestica*. The non-synonymous rate (Ka), synonymous rate (Ks), and evolutionary constraint (Ka/Ks) between the duplicated pairs of FaLysM proteins were computed using KaKs_Calculator (v2.0) (Zhang et al., 2006).

### Transcription factor binding sites of *FaLysM* genes predication

PlantRegMap (http://plantregmap.cbi.pku.edu.cn/binding_site_prediction.php) was used to search for the TF-binding sites within a 2000-bp genomic DNA sequence upstream of the transcriptional start site as the promoter sequence, with E values ≤ 1e−4 and 12 as the maximum motif number.

### Transcriptome data analysis

To identify the expression profiles of *FaLysM* genes during *C. fructicola* infection, the previously reported RNA-seq data from strawberry leaves infected with *C. fructicola* at 24, 72, and 96 hpi (hours post inoculation) were used. The RNA-seq data (the SRA accession number: SRP097590 and SRP099166) were reanalyzed on the basis of the newly updated genomic data of octoploid strawberry. Briefly, the software fastp was used to remove the reads containing adaptor contamination, low quality, or undefined bases. The valid reads were then mapped to the genome of octoploid strawberry using the software HISAT2. Subsequently, the software StringTie was used to assemble and estimate the expression levels of the transcripts (FPKM value), as previously described (Zhang et al., 2018).

### Fungal strains and plant material culture conditions

The WT strain of *C. fructicola* CGMCC3.17371, which was used in this study, was maintained on potato-dextrose agar (PDA) at 28° in the dark. The knockout mutant of the *CfLysM2* gene (*ΔCfLysM2*) (Yu et al., 2022) was maintained on PDA amended with 100 μg/mL hygromycin (Sangon Biotech). The strawberry (*Fragaria* × *ananassa* Duch.) cultivar ‘Jiuxiang’ was used in this study. The healthy stolon-derived ramets were maintained in a growth chamber at 22–25° and 70% humidity, with a light-dark cycle of 12/12 hr.

### Infection treatment, RNA extraction, and quantitative real-time polymerase chain reaction analysis

The healthy stolon-derived ramets (10 fully expanded compound leaves) were inoculated with a *C. fructicola* WT strain and *ΔCfLysM2* conidia suspension (10^6^ conidia/mL sterile water solution containing 0.01% Tween) in a growth chamber (Zhang et al., 2018). Mock inoculations of the ramets were performed using the Tween 20 water solution alone. The mature 5th−6th trifoliate leaves were harvested at 24, 72, and 96 hpi for quantitative real-time polymerase chain reaction (qRT-PCR) analysis.

qRT-PCR was performed as described by Zhang et al. (2018). Briefly, total RNA was extracted from strawberry leaf samples, which included a mock inoculation and inoculation of the leaf samples at 24, 72, and 96 hpi using RNAiso Plus (TaKaRa, Otsu, Japan), followed by reverse transcription with an oligo(dT) primer using the Superscript III RT-PCR kit (Invitrogen, Carlsbad, CA). The QuantStudio 1 Plus Real-Time PCR System (Thermo Fisher Scientific, Waltham, MA) was used for the qRT-PCR reactions with the 2×T5 Fast qPCR Mix (SYBR Green I, Singke Biotechnology Co., Ltd., Beijing, China) to amplify a final volume of 20 μL. Independent runs were repeated thrice for all reference and selected genes for each plate. The internal reference was the strawberry *Fagapdh* gene (Schaart et al., 2002). Reactions were performed in an Applied Biosystems QuantStudio 1 Plus qRT-PCR system (Thermo Fisher Scientific) with the following settings: 95°C for 2 min and 40 cycles of 95°C for 15 s and then 60°C for 20 s. The results were normalized with the internal reference genes. The 2^−ΔΔCt^ method was used to calculate the relative expression levels. The data were analyzed using analysis of variance, with *p* values < 0.05 considered statistically significant. The specific *FaLysM* primers were designed using the Primer 5.0 software and listed in **Supplementary Table S2**.

## Results

### Identification and characteristics of the sequenced octoploid strawberry LysM proteins

Based on the published octoploid strawberry genome (Edger et al., 2019), a total of 14 *LysM* genes were identified and confirmed with the LysM domain by using Pfam A (v33.1) and pfamscan (v1.6) database, named FaLysM1~FaLysM14 (**Supplementary Table S3**). The physicochemical characteristics of the FaLysM proteins were investigated using the ProtParam tool. The length of the 14 FaLysM protein sequences ranged from 298 (FaLysM9) to 978 amino acids (FaLysM4). The molecular weights of the LysM proteins ranged from 32.17 to 104.92 kDa, with isoelectric points (*pI*) ranging from 4.72 to 8.81 (**Table 1**). According to the *pI* values, most proteins (10 members, 71.4%) showed an acidic characteristic. The hydropathicity (GRAVY) index ranged from −0.10 (FaLysM1) to 0.41 (FaLysM2). One LYP member (FaLysM9) had the highest GRAVY score, showing a hydrophobic characteristic. In addition, most LysM proteins (10 members, 71.4%) exhibited a hydrophobic nature (**Table 1**). The prediction of the subcellular localization results showed 4 extracellular (secreted) proteins, 7 plasma membrane proteins, and 1 cytoplasm proteins.

**Table 1:** Features of octoploid strawberry LysM proteins (FaLysM).

Name	Gene ID	Molecular weight(Da)	Isoelectric points	Instability Index	Aliph-atic index	GRAVY	Localization	Singal peptide	Trans-membrane domain
FaLysM1	augustus_masked-Fvb6-2-processed-gene-280.2	52320.46	8.81	46.27	98.54	-0.103	Cytosol	No	1
FaLysM2	maker-Fvb2-1-augustus-gene-175.45	42568.64	6.09	48.89	97.18	0.182	Plasma membrane	Yes	3
FaLysM3	maker-Fvb2-1-augustus-gene-99.35	78786.43	6.31	46.71	96.18	-0.056	Plasma membrane	Yes	10
FaLysM4	maker-Fvb2-2-augustus-gene-102.36	72966.56	6.08	36.11	91.65	-0.048	Plasma membrane	Yes	10
FaLysM5	maker-Fvb2-2-augustus-gene-39.65	78568.10	6.11	46.29	96.18	-0.041	Extracellular	Yes	0
FaLysM6	maker-Fvb2-2-snap-gene-112.57	61806.61	5.24	35.43	93.79	-0.04	Plasma membrane	Yes	10
FaLysM7	maker-Fvb2-3-augustus-gene-18.41	50645.69	6.36	39.8	102	0.414	Extracellular	Yes	0
FaLysM8	maker-Fvb2-4-augustus-gene-195.56	104947.44	8.46	38.78	98.61	0.311	Extracellular	Yes	0
FaLysM9	maker-Fvb5-3-snap-gene-212.56	104918.25	8.31	40.08	98.13	0.304	Chloroplast	Yes	0
FaLysM10	maker-Fvb5-4-augustus-gene-61.63	35028.15	5.22	31.42	95.78	0.288	Extracellular	Yes	1
FaLysM11	maker-Fvb6-1-augustus-gene-12.58	98873.35	8.34	39.83	99.86	0.299	Plasma membrane	No	3
FaLysM12	maker-Fvb6-2-augustus-gene-39.17	35009.06	5.07	31.28	98.13	0.292	Plasma membrane	Yes	2
FaLysM13	maker-Fvb6-3-augustus-gene-11.53	34921.93	4.72	29.13	98.4	0.319	Plasma membrane	No	3
FaLysM14	maker-Fvb6-4-augustus-gene-243.39	32168.18	6.28	43.06	105.94	0.326	Cytosol	Yes	0

### Protein model analysis of the FaLysM proteins

**Figure 1:**
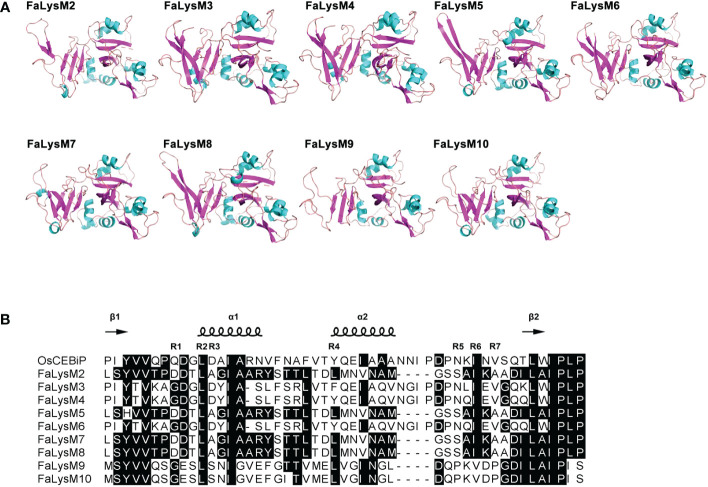
Analysis of structural models of FaLysM proteins by Pymol. **(A)** The structural models of FaLysM proteins. Different colors represent different secondary structures (cyan:helix; magenta: beta-sheet; pink: loop). **(B)** Sequence alignment of conserved mode of NAG recognition by LysM proteins between OsCEBiP (LysM2 domain, amino acids 111-161) and FaLysMs. R1-R7 indicate the conserved position in the secondary structures. Consensus and similar amino acid residues for all sequences are shown in solid black boxes.

The amino acids of the FaLysM proteins were uploaded in Phyre^2^ for protein homology analysis and in PyMol for modeling construction. The results showed that nine FaLysM proteins (FaLysM2-10) had high similarities to protein OsCEBiP (5jce in rice), all with high confidence rates (100%) (**Figure 1A**). A highly conserved mode, including 7 conserved residues (labeled R1–R7) from the LysM proteins, was reported for *N*-acetylglucosamine (NAG) recognition (Liu et al., 2016). Consistently, a high similarity of this mode was observed between FaLysM2-10 and OsCEBiP, indicating their potential function in NAG recognition. The residue I150 (R6) of OsCEBiP has a critical contribution to chitin binding, as confirmed by a mutation experiment. In our study, this residue is highly conserved in FaLysM proteins, except for FaLysM9 and FaLysM10 (**Figure 1B**).

### Phylogenetic analysis of LysM proteins

**Figure 2:**
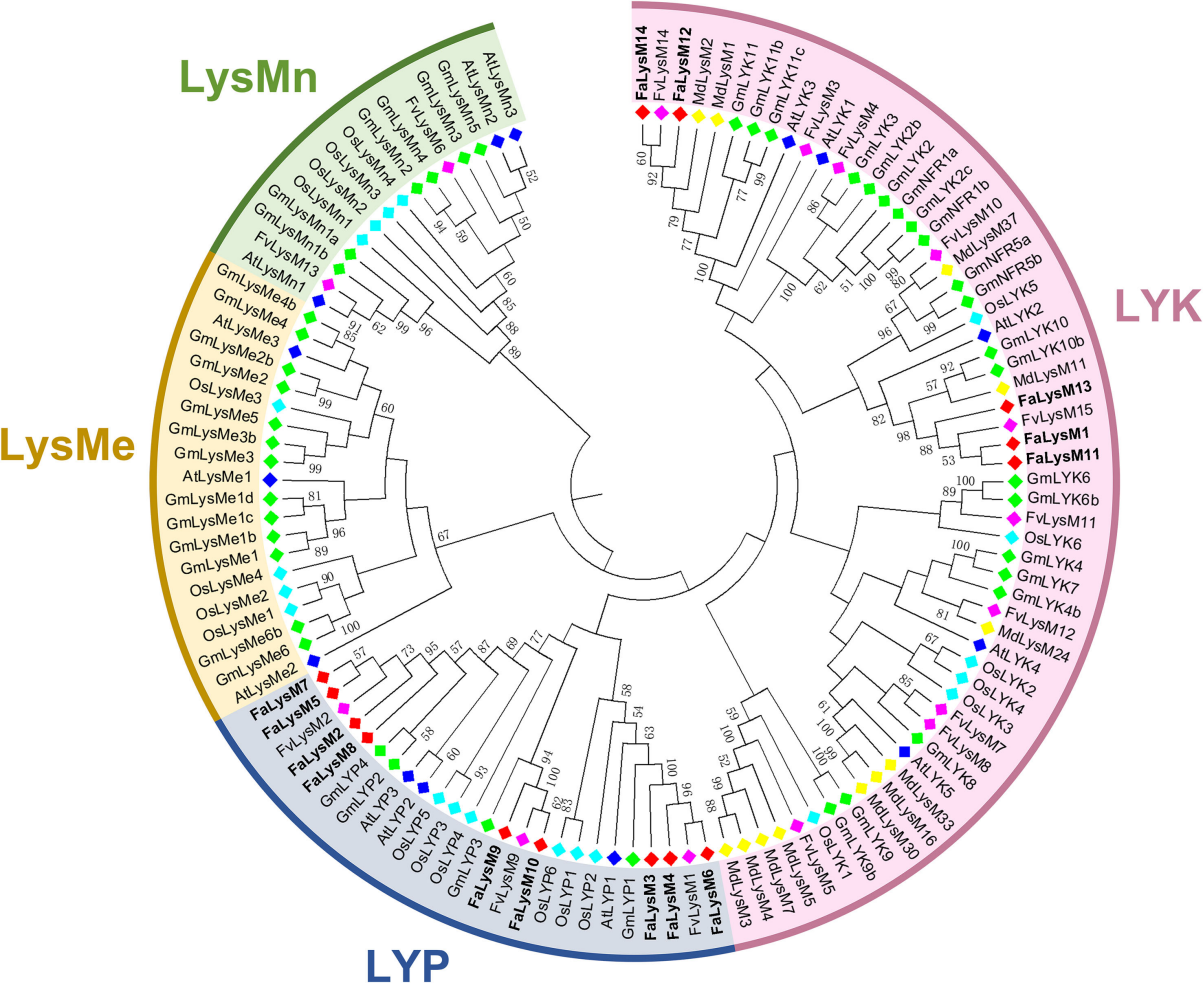
Phylogenetic relationships of LysM proteins in cultivated octoploid strawberry, wild strawberry, Arabidopsis, soybean, rice, and apple. LysM proteins from cultivated octoploid strawberry, wild strawberry, Arabidopsis, soybean, rice, and apple are labeled with the prefix ‘Fa’, ‘Fv’, ‘At’, ‘Gm’ ‘Os’ and ‘Md’, respectively. Different background colors indicate the different groups of the LysM proteins. The phylogenetic tree was constructed by MEGA-X with the neighbor-joining method.

To characterize the evolutionary relationships of the LysM protein family members, the LysM proteins in *Arabidopsis* (At), wild strawberry (Fv), soybean (Gm), rice (Os), and apple (Md) were sequenced, and a NJ tree was constructed with MEGA-X. The LysM proteins from all these plants were divided into four subfamilies: the LYK (I), LYP (II), LysMn (III), and LysMe subfamilies (IV). The LYK and LYP subfamilies could be identified in the octoploid Strawberry. Among them, the LYK and LYP subfamilies have 5 and 9 members, respectively. AtCERK1, AtLYK4, and AtLYK5 were proved to recognize chitin. Although no FaLysM proteins were found to form a monophyletic cluster with the three LYK subfamilies, FaLysM1 and FaLysM11 showed a relatively close relationship with AtLYK4 and AtLYK5, indicating their role as putative receptors that recognize chitin (**Figure 2**).

### Gene structural analysis and conserved motif identification of FaLysM proteins

**Figure 3:**
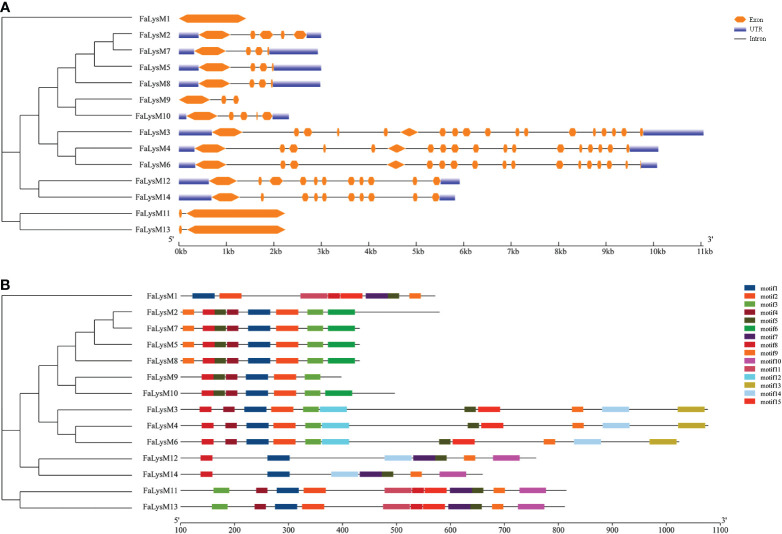
The conserved motifs and exon-intron structure of LysM genes in octoploid strawberry. **(A)** Gene structure of *FaLysM genes*. Exons and introns were represented by orange boxes and black lines, respectively. And the genomic length is indicated at the bottom. **(B)** Motif composition of *FaLysM genes*. Different colored boxes indicate motifs 1-15, and the information for each motif is in Supplementary Table S5. The scale of the length of proteins is at the bottom.

The intronic numbers of *FaLysM* genes ranged from 0 to 17, with an average of 6. The *FaLysM3* and *FaLysM4* genes of the LYP family members contained 17 introns, which were the longest introns. Only one gene (*FaLysM1*) lacked intronic structures. Most *FaLysM* genes exhibited one, three, or four forms of intron splicing. The exons ranged from 1 to 18 in FaLysM. *FaLysM3* and *FaLysM4*, which were the most diverse in terms of the number of exons, were observed in the LYP subfamily (**Figure 3A**, Supplementary Table S4).

According to our data, 15 conserved motifs have been discovered, although the lowest number of motifs (6 motifs) was detected in FaLysM9 (**Figure 3B**). The highest number of motifs were related to FaLysM3, FaLysM4, FaLysM6, FaLysM11, and FaLysM13 with 11 motifs, followed by FaLysM1, FaLysM2, FaLysM7, and FaLysM8 with 8 motifs. Each subgroup showed approximately similar motif compositions. The number of amino acids in each conserved motif ranged from 20 to 49 (**Supplementary Table S5**). All FaLysM proteins contained motifs 1, 5, and 8. Motifs 2, 3, and 4 were usually found together in most FaLysM proteins.

### Chromosomal distribution and collinearity analysis of duplicated *FaLysM* genes

**Figure 4:**
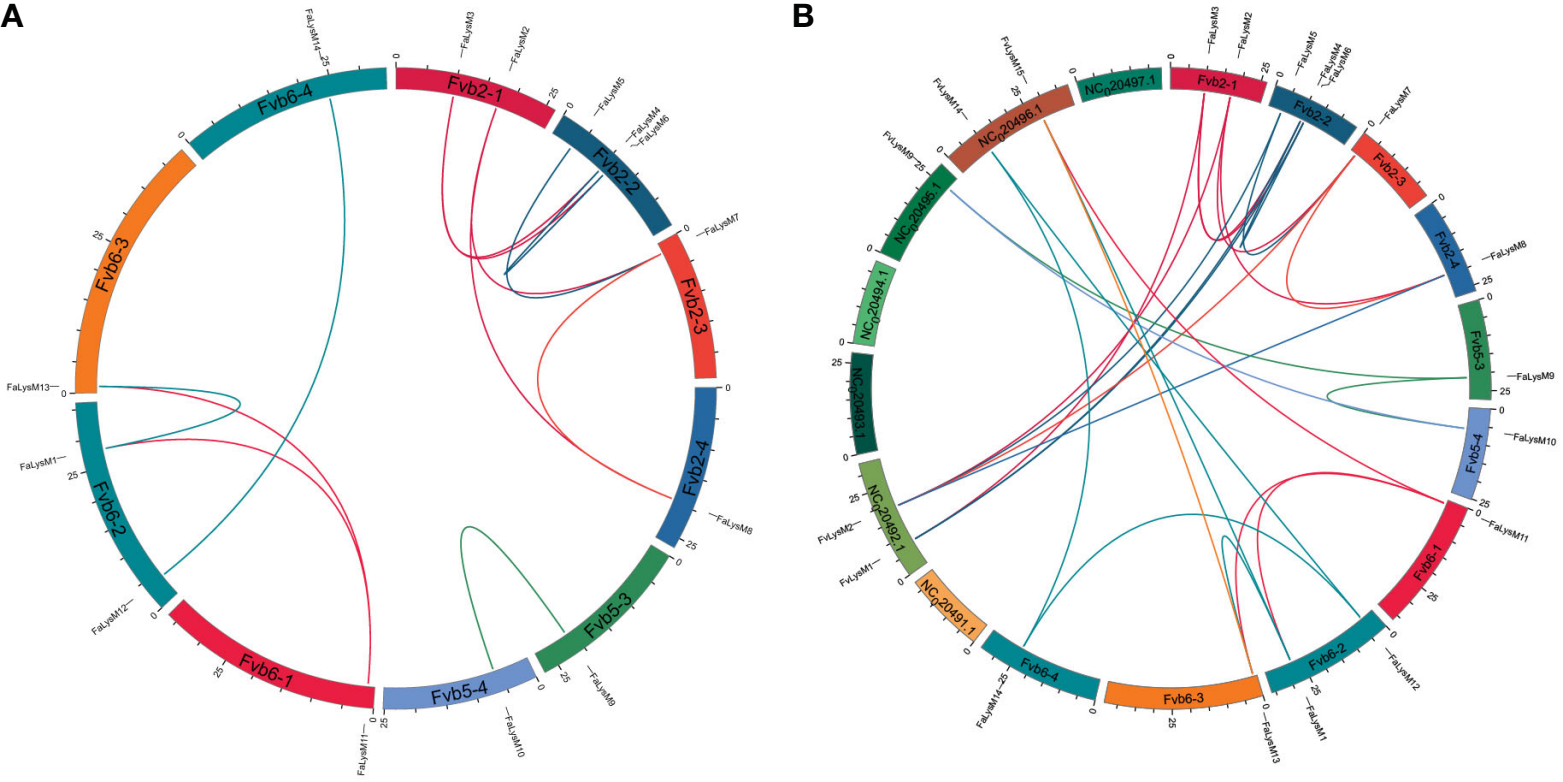
Collinearity analyses of *FaLysM* genes in the F. × ananassa genome, and between *F. × ananassa* and *F. vesca*. **(A)** Collinearity analysis of *FaLysM* genes in *F. × ananassa*; **(B)** Interspecific collinearity analysis of LysM genes between *F. × ananassa* and *F. vesca*; Outer boxes represented chromosome numbers. Colored lines in boxes indicated the location of LysM genes in each chromosome. Gene pairs with a syntenic relationship are joined by colored lines.

The chromosomal location analysis revealed that the *FaLysM* genes in *F*. × *ananassa* were unevenly distributed on 10 chromosomes. *FaLysM1* and *FaLysM12* were located on chromosome Fvb6-2; *FaLysM2* and *FaLysM3*, on chromosome Fvb2-1; *FaLysM4*, *FaLysM5*, and *FaLysM6*, on chromosome Fvb2-2; and *FaLysM7*, *FaLysM8*, *FaLysM9*, *FaLysM10*, *FaLysM11*, *FaLysM13*, and *FaLysM14*, on chromosomes Fvb2-3, Fvb2-4, Fvb5-3, Fvb5-4, Fvb6-1, and Fvb6-3, respectively (**Supplementary Figure S1**). On the basis of a genome-wide analysis of gene duplications, 12 pairs of genes were identified as duplication genes into chromosomes (**Figure 4A**; Supplementary Table S6). The Ka/Ks ratio represents the selection intensity and direction. In addition to FaLysM4/FaLysM6, the Ka/Ks values of the remaining FaLysMs genes were <1, except for one paired gene (FaLysM4/FaLysM6), which had a Ka/Ks ratio of >1 (**Supplementary Table S6**). The interspecific synteny between *F*. × *ananassa* and *F*. *vesca* was compared and analyzed to further examine the evolution of *FaLysMs* genes (**Figure 4B**). The results showed that 14 gene pairs had syntenic relationships with *F*. × *ananassa* and *F*. *vesca*. The Ka/Ks values of all the syntenic gene pairs were less than 1, indicating that they might have experienced purifying selection (**Supplementary Table S7).**

### Transcription factor binding sites analysis in the FaLysM promoters

**Figure 5:**
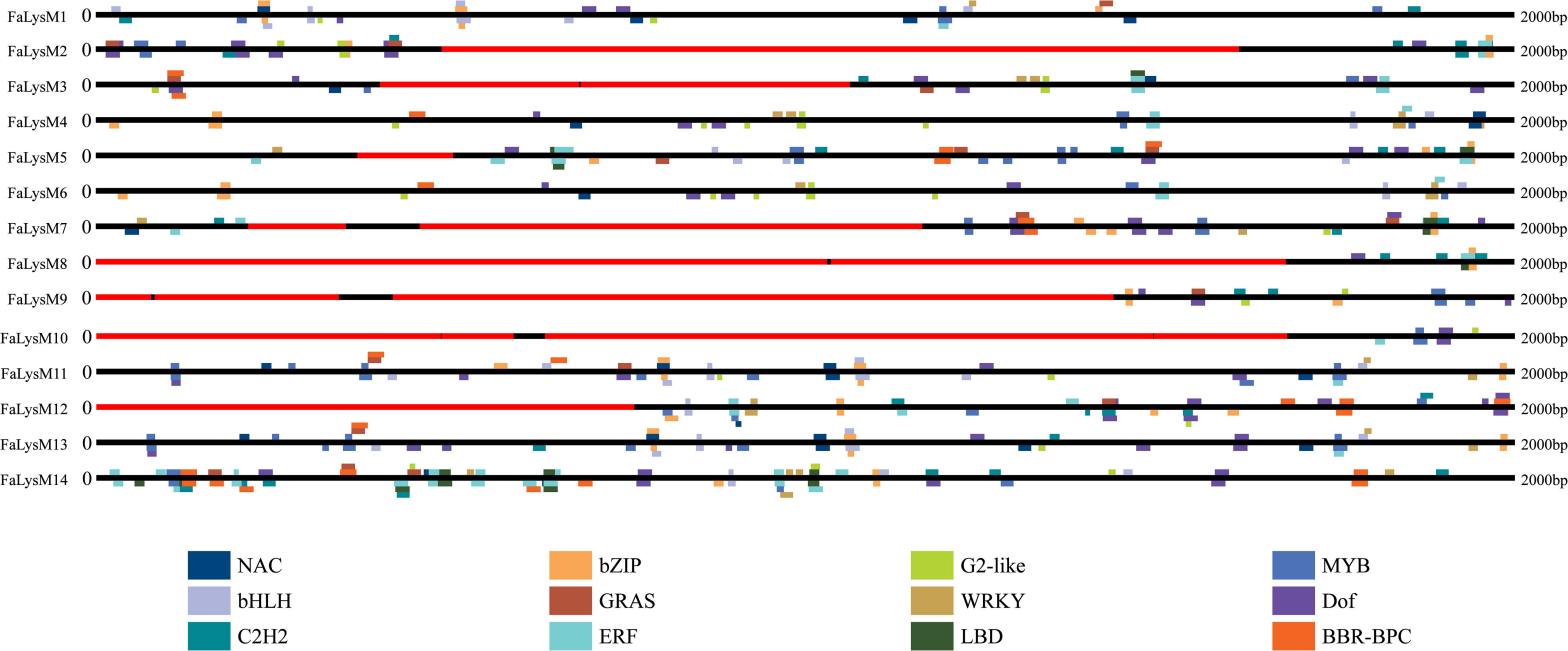
Distribution of transcription factor binding sites in the promoter of FaLysM genes. Twelve different colors represent twelve different transcription factors.

To examine the possible binding sites of TFs, the 2-kb upstream promoter regions of the TaASRs were examined using the online database PlantRegMap. The results showed that a total of 479 binding cites for 12 TFs, including NAM/ATAF/CUC (NAC) (Nakashima et al., 2007; Shao et al., 2015), BARLEY B-RECOMBINANT/BASIC PENTACYSTEINE (BBR/BPC), WRKY, ethylene response factor (ERF), Cys2His2 (C2H2), DNA binding with one finger (Dof), basic leucine zipper (bZIP), basic helix-loop-helix (bHLH), lateral organ boundaries domain (LBD), myeloblastosis (MYB), GRAS, and G2-like, were discovered. The Dof-binding sites occurred 70 times, and they were present in the promoter of all 14 *FaLysM* genes. We identified 51, 60, and 61 binding sites of ERF, bZIP, and MYB, respectively, spanning 13 *FaLysM* promoters, which were absent from *FaLysM9*, *FaLysM3*, and *FaLysM8*, respectively. Most of these TFs, such as NAC, WRKY, ERF, C2H2, bZIP, bHLH, LBD, MYB, and G2-like, were involved in the regulation of plant growth and development processes, including abiotic stress responses such as pathogen defense (**Figure 5**; **Supplementary Table S8**).

### Identification of differentially expressed *FaLysM* genes in response to *Colletotrichum fructicola* in Octoploid Strawberry leaves

**Figure 6:**
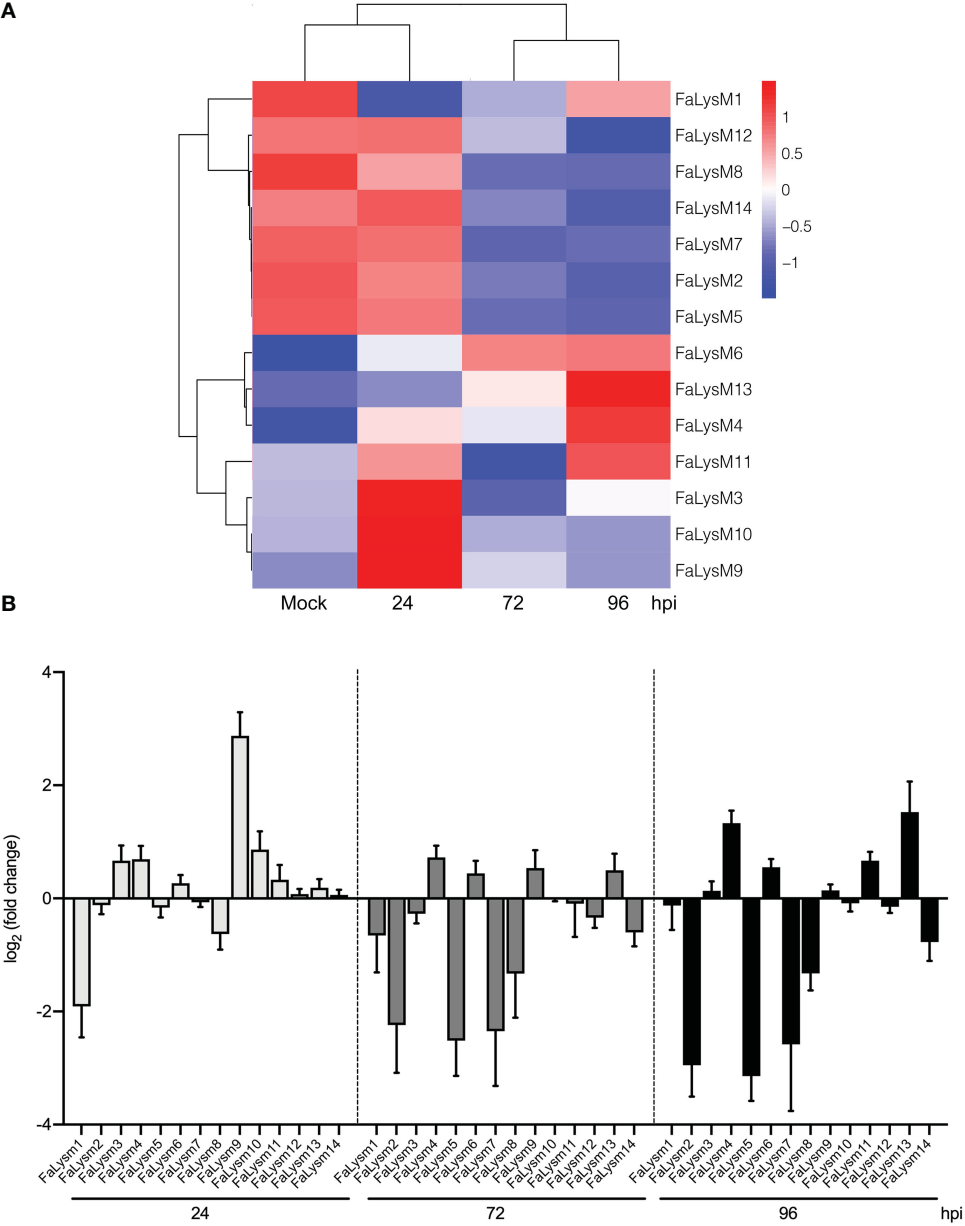
The expression patterns of *FaLysM* genes in octoploid strawberry leaves infected with *C. fructicola* at different stages (24, 72 and 96 hpi). **(A)** The heat map analysis of 14 differentially expressed *FaLysM* genes in strawberry leaves infected with *C. fructicola* at 24, 72 and 96 hpi. Mock: Mock treatment with water containing Tween -20. **(B)** The relative expression levels of *FaLysM* genes in strawberry leaves infected with *C. fructicola* at 24, 72 and 96 hpi. Error bars represent the standard error of the mean. x-axis shows genes in three time points validated in this study; y-axis shows the log2 ratio of *FaLysM* gene expression in infected strawberry (24, 72 and 96 hpi) versus mock inoculated strawberry.

Three *C*. *fructicola* infection stages (24, 72, and 96 hpi) in octoploid strawberry leaves were used for transcriptome profiling. Compared with the data from genome-wide analysis of the *FaLysM* gene family, the transcriptome data showed that the *FaLysM* genes were differentially expressed (**Figure 6A**; **Supplementary Table S9**). The expression profiles of these *FaLysM* genes were further validated by RT-qPCR (**Figure 6B**). The trends in expression levels of 14 *FaLysM* genes were similarto those found in the RNA-seq data. These differentially expressed genes (DEGs) exhibited differential expressions across three *C*. *fructicola* infection stages. The expression profiles could be simply divided into four categories: (1) upregulation at the early stage of infection (*FaLysM3*, *FaLysM9*, and *FaLysM10*); (2) upregulation in the late stage of infection (*FaLysM4*, *FaLysM6*, *FaLysM11*, and *FaLysM13*); (3) gradual downregulation (*FaLysM2*, *FaLysM5*, *FaLysM7*, *FaLysM8*, *FaLysM12*, and *FaLysM14*); (4) downregulation in the early stage of infection (*FaLysM1*). These results indicate the differential regulation of *FaLysMs* by *C*. *fructicola* infection.

**Figure 7:**
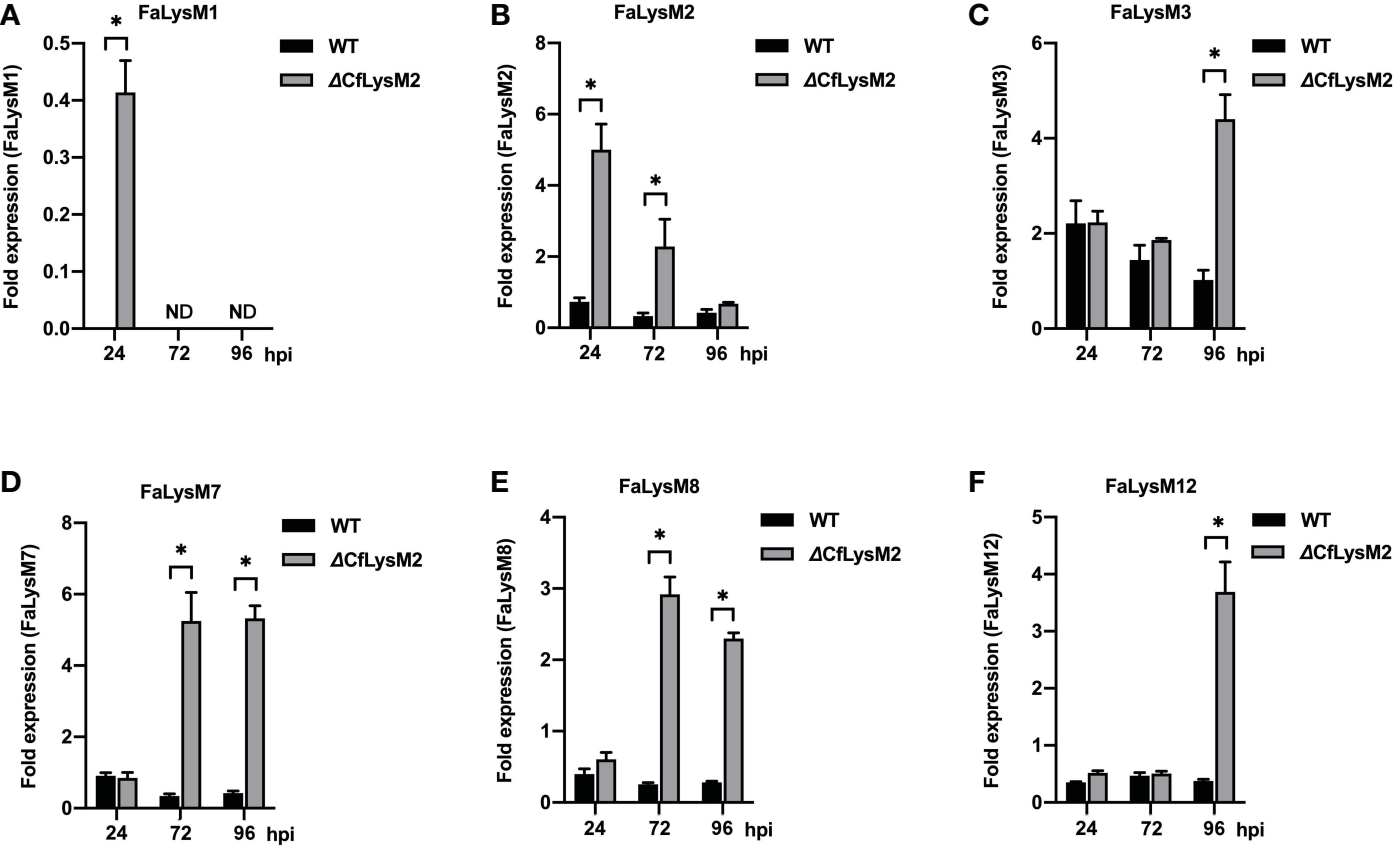
The relative expression levels of *FaLysM* genes in strawberry leaves infected with WT or Δ*CfLysM2* of *C. fructicola*, respectively. Samples were collected at different time points (0, 24, 72, 96 hpi) for qRT-PCR analysis with *FaLysM1* **(A)**, *FaLysM2* **(B)**, *FaLysM3* **(C)**, *FaLysM7* **(D)**, *FaLysM8* **(E)**, or *FaLysM12* **(F)** primers, respectively. Y-axis shows the ratio of *FaLysM* gene expression in infected strawberry (24, 72, and 96 hpi) vs. mock inoculated strawberry. * Indicate *P* ≤ 0.05 (Student’s t test).

To elucidate whether the *C*. *fructicola* LysM effector (*CfLysM2*) functions in the regulation of strawberry LysM protein family (FaLysM)-mediated immunity, benihoppe leaves were infected with WT and Δ*CfLysM2* of *C*. *fructicola* and collected at different time points (0, 24, 72, and 96 hr) and subjected to qRT-PCR analysis to evaluate the expression profiles of all 14 *FaLysM* genes. The results showed that the mutation of *CfLysM2* leads to the higher expression of six *FaLysM* genes (*FaLysM1*, *FaLysM2*, *FaLysM3*, *FaLysM7*, *FaLysM8*, and *FaLysM12*) at different time points, compared with those during WT strain infection (**Figure 7**). The expression levels of other *FaLysM* genes showed no significant differences between the WT strain and *ΔCfLysM2* infection groups (data not shown). Collectively, these results demonstrated extensive reactions of the *FaLysM* genes in response to *C*. *fructicola* infection and the potential role of the effector gene *CfLysM2* in the regulation of expression of various *FaLysM* genes.

## Discussion

Proteins containing the LysM motif were proved to be widely distributed in almost all organisms, including eukaryotic and prokaryotic organisms (Buist et al., 2008). In plants, an earlier study confirmed the classical chitin-binding function of one LysM protein family (CEBiP) (Kaku et al., 2006). Since then, proteins containing the LysM motif have been identified in many plants such as *Arabidopsis* (16), wild strawberry (*F*. *vesca*) (n=11), rice (n=15), wheat (n=60), sweet orange (n=9), and *Brachypodium distachyon* (n=11) (Shiu et al., 2004; Zhang et al., 2009; Zhou et al., 2014; Tombuloglu et al., 2019; Chen et al., 2020; Liu et al., 2020; Li et al., 2021). Previous reports have concluded no meaningful relationship between genome size and the number of genes in plants (Abedi et al., 2021). The observed difference in the number of LysM genes identified in this study may be due to differences in detection criteria. In the present study, 14 LysM proteins were found in octoploid strawberry. The identified FaLysM proteins were categorized into LYK and LYP groups. These proteins were unevenly distributed on 10 chromosomes in the octoploid genome. It should be noted that previous studies identified two LysM proteins containing only one LysM domain in wild strawberry and defined as LysMn proteins (Liu et al., 2020). The LysMn proteins were not identified in our study, indicating the LysMn genes were probably lost or missed in the assembled genome, which may explain the relative disease resistance of wild strawberry to some extent.

Gene duplication events lead to increases in the number of plant genes, which drive genome evolution, in which the creation of new genes is often driven by selective pressure (Murat et al., 2012; Huang et al., 2022). The Ka/Ks ratio is an important index for evaluating evolution across or within the same species (Sironi et al., 2015). In our study, the Ka/Ks ratios of most *FaLysM* genes were <1, which indicates that its selection pressure was mostly subject to purifying selection during evolution, consistent with the results from banana, wild tomato, and the allotetraploid *Brassica napus* L (Richards and Rose, 2019; Abedi et al., 2021; Ren et al., 2022). These results suggest that most nonsynonymous substitutions were harmful and were thus eliminated during evolution. The Ka/Ks ratio for one paired gene (*FaLysM4*/*FaLysM6*) was >1, indicating positive selection, probably resulting in multiple functions as a result of mutations during their evolution.

Owing to the high purifying selection in the *LysM* gene family, the importance of the functional role of *FaLysM* genes has been determined. The phylogenetic tree showed that FaLysM proteins have a close relationship with their counterparts, indicating that the phylogenetic distribution of FaLysM protein is associated with their motif contents. All members of the FaLysM protein family contain 3 common motifs, motifs 1, 5, and 8. Most FaLysM proteins contain motifs 2, 3, 4, and 9. The difference between the members of this subfamily was related to (1) motif 6 being exclusively found in the group of LYPs (FaLysM2, FaLysM5, FaLysM7, FaLysM8, and FaLysM10); (2) motif 7 being exclusively fond in the group of LYKs (FaLysM1, FaLysM11, FaLysM12, FaLysM13, and FaLysM14); (3) motif 10 being exclusively found in the group of LYKs (FaLysM11, FaLysM12, FaLysM13, and FaLysM14); (4) motif 11 being in three types of LYKs (FaLysM1, FaLysM11, and FaLysM13); and (5) motifs 12 and 13 being in three types of LYPs (FaLysM3, FaLysM4, and FaLysM6). These results are completely consistent with the results of the phylogenetic tree. The prediction of the subcellular localization results showed 3 LYPs, namely FaLysM3, FaLysM4, and FaLysM6, all containing up to 10 transmembrane domains, which suggests the possibility that they may act as surface-localized receptors recognizing evading pathogens, consistent with the previous study that showed that CEBiP is a plasma membrane-localized glycoprotein binding to chitin and subsequently inducing downstream immune responses (Kaku et al., 2006).

The phylogenetic analysis of LysM proteins from different plants can provide insights into their potential roles. Chitin elicitor receptor kinase 1 of *Arabidopsis* (AtCERK1 and AtLYK1), as a critical cell surface receptor, directly binds chitin through the LysM ectodomain to initiate immune responses (Liu et al., 2012b). Overexpression of AtLYK2 enhanced tolerance to fungus and increased the expression levels of defense-related genes during infection (Giovannoni et al., 2021). In our study, a relatively close relationship was observed between AtLYK1 and FaLysM12/FaLysM14, and between AtLYK2 and FaLysM1/FaLysM11/FaLysM13, which suggests that they may also mediate chitin signaling processes. AtLYP1 and AtLYP3 directly interact with PGN and mediate the immunity to bacterial infection (Willmann et al., 2011). From the phylogenetic tree, FaLysM3/FaLysM4/FaLysM6 and AtLYP1 are in the same clade, while other FaLysM proteins and AtLYP3 are in the same clade, which suggests their possible roles in PGN-mediated immunity to bacterial infection. LYKs formed many distinct clades constructed by branches with a single gene, compared with LYPs, possibly indicating that LYKs have undergone faster evolution and maintained relatively more diversity in sequence than LYPs. This phenomenon could also be observed in the NLR family, of which the CNL family is highly diverged compared with the TNL family (Wan et al., 2013; Alamery et al., 2017).

The TFs play an important role in response to environmental stresses by binding to the promoters of the target genes and regulating their expressions (Lindemose et al., 2013). The TF-binding site analysis results suggested that *FaLysM* genes were involved in various processes during growth and development. As expected, most TFs were associated with plant resistance against biotic stresses, such as NAC, WRKY, ERF, C2H2, bZIP, bHLH, LBD, MYB, and G2-like.

The protein modeling analysis of FaLysM proteins provided insights into their regulation of pathogen-plant interactions. As the first identified chitin oligosaccharide receptor in plants, OsCEBiP is a typical LysM domain-containing protein with two LysMs in the extracellular domain at the N terminus and a transmembrane domain at the C terminus (Kaku et al., 2006). The crystal structures of a free ectodomain of OsCEBiP (OsCEBiP-ECD) in complex with the chitin tetramer (NAG)4 demonstrated that three LysM domains pack tightly against each other, and only the LysM2 domain binds to chitin (Liu et al., 2016). As demonstrated in both plants and fungi, various LysM domain-containing proteins are known to interact with chitin (De Jonge et al., 2010; Sánchez-Vallet et al., 2013; Sanchez-Vallet et al., 2015). A highly conserved mode, including seven conserved residues from the LysM proteins of several plants and fungi, was reported for chitin recognition (Liu et al., 2016). In this study, this mode, especially the several critical residues such as I150 (R6), was also observed in as many as seven FaLysM proteins, demonstrating the potential role of FaLysM proteins in chitin recognition.

PAMPs are defined as highly conserved microbe-specific molecules recognized by PRRs and therefore have an essential function in plant survival or fitness (Jones and Dangl, 2006; Yuan et al., 2021). The expression profiles of these PRR genes provide valuable information on their functions in anti-pathogen plant immunity. Our previous transcriptomic data provides a picture of how strawberry mobilized plant immune system in response to *C. fructicola* infection, including the differential regulation of several PRR genes (Zhang et al., 2018). In this study, we reanalyzed the RNA-Seq data and focused on *FaLysM* genes to obtain their expression profiles in response to *C. fructicola* infection. The results showed the differential regulation of different *FaLysM* genes in response to *C. fructicola* infection, implying the complex interaction between *C. fructicola* and strawberry.

Some PRR genes such as *FaLysM4*, *FaLysM6*, *FaLysM11*, and *FaLysM13* have peak expression levels at the late stage of infection (96 hpi), but their early expression levels are not significantly affected, which indicates that although these *FaLysM* genes were ultimately upregulated at the stage when the leaves developed visible anthracnose symptoms, they appeared to be not activated initially after *C. fructicola* infection. Another notable example is the significant downregulation of *FaLysM1* at 24 hpi upon the onset of *C. fructicola* infection. Except for these situations, the dominant expression profile is the upregulation of *FaLysM* genes such as *FaLysM3*, *FaLysM9*, and *FaLysM10* at the early stage of infection, which indicates that these PRR genes were rapidly stimulated after infection. However, these PRRs, together with some other PRR genes such as *FaLysM2*, *FaLysM5*, *FaLysM7*, *FaLysM8*, *FaLysM12*, and *FaLysM14*, were downregulated at the late stage of infection. A recent study also showed the elevated expression levels of candidate *LysM* genes at 6 to 24 hpi, followed by a decrease at 48 hpi, in *Coffea arabica* infected with *Hemileia vastatrix* (Santos et al., 2022). These results describe that most PRR genes exhibit an initial stimulus with subsequent suppression upon fungus infection. Collectively, the distinctive expression profiles of the *FaLysM* genes are reminiscent of the complex and competitive relationship between the *CfLysM2* and *FaLysM* genes, just like the similar expression profiles of other PRRs in our previous results, such as leucine-rich repeat receptor-like protein kinases (LRR-RLKs) (Zhang et al., 2018).

Various studies have demonstrated the interference of chitin-mediated plant immunity by LysM effectors elicited by different fungi (De Jonge and Thomma, 2009; Marshall et al., 2011; Mentlak et al., 2012). A typical example is the sequestration of chitin by Ecp6, a secreted LysM effector of the fungus *Cladosporium fulvum*, to perturb chitin-induced tomato immunity (De Jonge et al., 2010). A similar function was also proved for *Magnaporthe oryzae* Slp1 and *Mycosphaerella graminicola* Mg3LysM (Marshall et al., 2011; Mentlak et al., 2012). The inhibition of PRR expression may be associated with the suppression of PTI signaling (Santos et al., 2022). From our transcriptome data, we chose the significantly upregulated LysM effector gene *CfLysM2* during *C*. *fructicola* infection, to generate the knockout mutant *CfLysM2*. By comparing the expression profiles of the *FaLysM* genes in response to both WT and Δ*CfLysM2*, we conclude that the inhibition of most *FaLysM* gene expressions by *CfLysM2* is reminiscent of the possible competition between *C*. *fructicola* LysM effectors and FaLysM proteins.

## Conclusions

In this study, 14 FaLysM protein members were identified in octoploid strawberry, including 5 LYKs and 9 LYPs. The protein model analysis results showed that nine FaLysM proteins contain a conserved mode of chitin binding, which suggests their potential roles in pathogen perception and plant immunity. The distinct expression profiles of the *FaLysM* genes upon the onset of *C*. *fructicola* infection suggest the complex interaction between *C*. *fructicola* and strawberry. By comparing the expression profiles of the *FaLysM* genes upon the onset of *C*. *fructicola* WT and Δ*CfLysM2* infections, we found that most *FaLysM* gene expressions are broadly inhibited by *CfLysM2* infection. These findings provide fundamental information on the strawberry LysM proteins and their interactions with *C*. *fructicola*, which promotes in-depth investigations on the interaction between *C*. *fructicola* and strawberry and on resistance breeding of strawberry.

## Data availability statement

The datasets presented in this study can be found in online repositories. The names of the repository/repositories and accession number(s) can be found in the article/**Supplementary Material**.

## Author contributions

The study was conceived by LZ, HA and XZ. LZ prepared the *C*. *fructicola* isolate and plant materials and performed the experiments. SL performed the bioinformatics analysis. LZ and XF performed the experiments of infection treatment, RNA Extraction, and qRT-PCR Analysis. LZ, HA and XZ prepared the manuscript. All authors contributed to the article and approved the submitted version.
