# Supplementary figures and images for: Genome-wide analysis of LysM gene family members and their expression in response to Colletotrichum fructicola infection in Octoploid strawberry(Fragaria × ananassa)
Source: Front Plant Sci. 2023 Jan 23;13:1105591. doi: 10.3389/fpls.2022.1105591 (PMC9900028; doi:10.3389/fpls.2022.1105591)

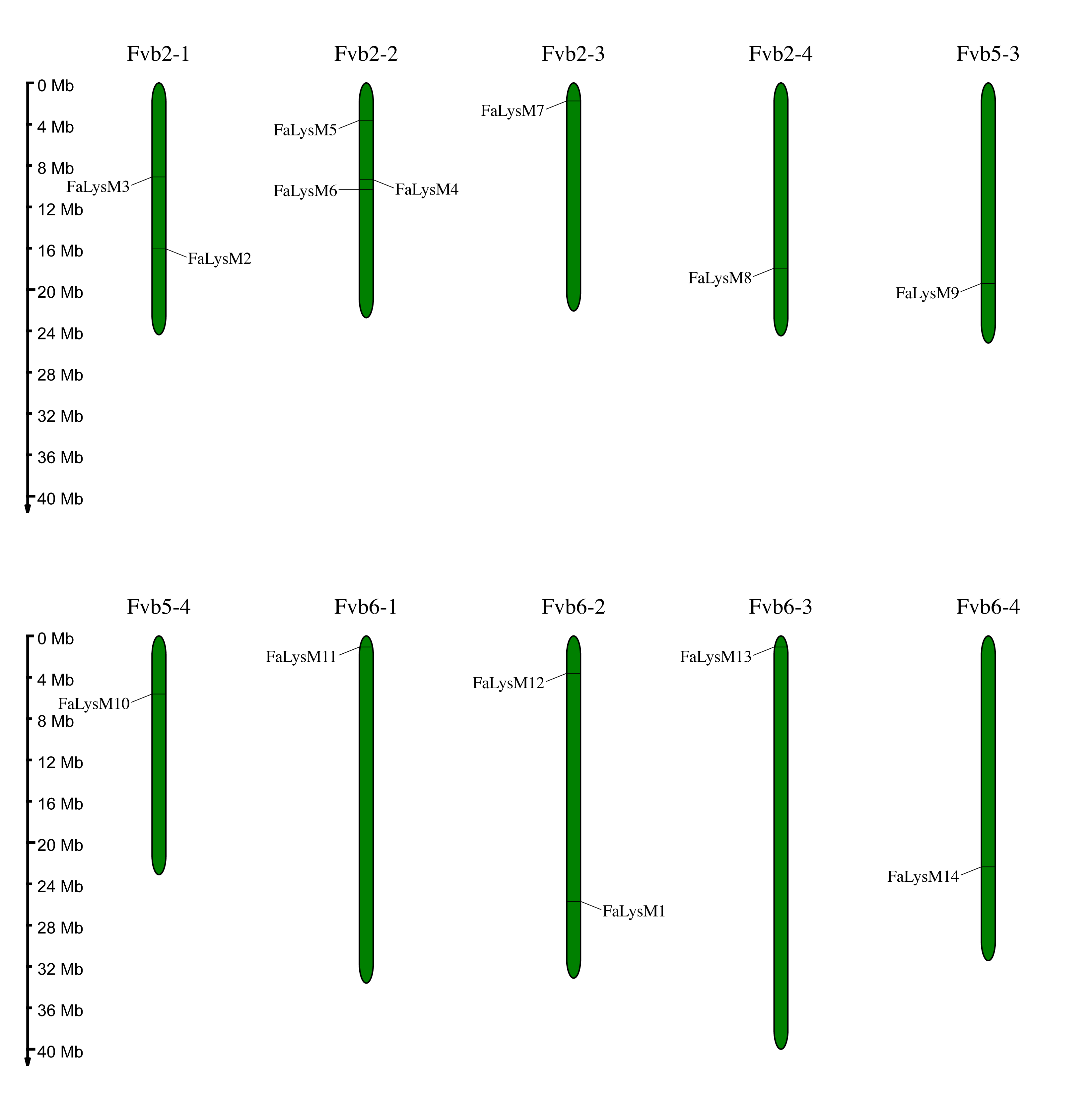

Supplement: Supplementary file 1 [file Image_1.tif]
